# T Cells Cause Lung Damage in Emphysema

**DOI:** 10.1371/journal.pmed.0010025

**Published:** 2004-10-19

T lymphocytes may have an important role in the pathogenesis of smoking-related emphysema, according to a new study by researchers from Houston, Texas, United States. “We now know that T cells are not only present in chronic obstructive pulmonary disease [COPD], but are harmful,” comments Steven Shapiro from Brigham and Women's Hospital**,** Harvard Medical School, who was not involved in the study. “We also now have a pathway that could be interrupted to prevent lung destruction in COPD.”

Farrah Kheradmand and colleagues took lung samples from 28 ex-smokers who had been admitted to hospital for lung resection: 18 patients had moderate to severe COPD as well as evidence of emphysema, and ten patients had none. The researchers isolated lung lymphocytes from the samples and used two-color flow cytometry to phenotypically characterize the cells. They found that lymphocytes taken from patients with emphysema expressed more CCR5 and CXCR3 receptors, which are associated with a particular type of T cell called T helper 1 (Th1), than did those from control individuals. By contrast, expression of CCR4 receptors, which are found on T helper 2 (Th2) cells, was very low in both control and emphysema groups.[Fig pmed-0010025-g001]

**Figure pmed-0010025-g001:**
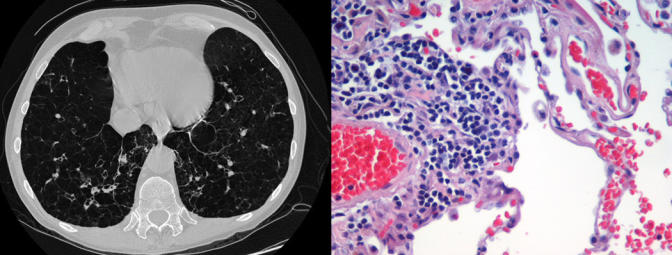
CT image of the lung of subjects with end-stage emphysema next to a photomicrograph of their resected lung stained with H&E

In a separate experiment, Kheradmand's team showed that lung lymphocytes taken from patients with emphysema secreted more of three other proteins—interferon gamma, monokine induced by interferon (MIG), and interferon-inducible protein 10 (IP-10)—than control patients. MIG and IP-10 are known to be produced by injured epithelial cells and are ligands for CXCR3 receptors, which are expressed by Th1 cells. Importantly, the researchers were also able to show that isolated peripheral lung macrophages secreted matrix metalloproteinase-12 (MMP12), an enzyme that degrades elastin—a protein important for lung elasticity—in the lungs, in response to IP-10 and MIG. Together these findings, say the authors, indicate that Th1 cells, but not Th2 cells, are required for producing the elastin-destroying lung environment of emphysema.

The researchers now intend to investigate the antigens that drive the Th1-based inflammation that underlies emphysema. “Ultimately, we seek to understand the biochemistry of tobacco smoke that triggers inflammation in the first place, and whether such insight might explain other environmentally triggered lung diseases,” explains Kheradmand. “To understand such detailed immune mechanisms, we really need an improved experimental model of disease, and this we are currently working on.”

